# β-arrestin-1 and β-arrestin-2 Restrain MRGPRX2-Triggered Degranulation and ERK1/2 Activation in Human Skin Mast Cells

**DOI:** 10.3389/falgy.2022.930233

**Published:** 2022-07-15

**Authors:** Zhao Wang, Zhuoran Li, Gürkan Bal, Kristin Franke, Torsten Zuberbier, Magda Babina

**Affiliations:** ^1^Fraunhofer Institute for Translational Medicine and Pharmacology ITMP, Immunology and Allergology IA, Berlin, Germany; ^2^Institute for Allergology, Charité-Universitätsmedizin Berlin, Corporate Member of Freie Universität Berlin, Berlin Institute of Health, Humboldt-Universität zu Berlin, Berlin, Germany; ^3^Department of Dermatology, The Second Affiliated Hospital, Northwest Hospital, Xi'an Jiaotong University, Xi'an, China

**Keywords:** MRGPRX2, mast cells, β-arrestin, skin, degranulation, signal transduction, ERK1/2

## Abstract

As a novel receptor that efficiently elicits degranulation upon binding to one of its numerous ligands, MRGPRX2 has moved to the center of attention in mast cell (MC) research. Indeed, MRGPRX2 is believed to be a major component of pseudo-allergic reactions to drugs and of neuropeptide-elicited MC activation in skin diseases alike. MRGPRX2 signals *via* G proteins which organize downstream events ultimately leading to granule discharge. Skin MCs require both PI3K and ERK1/2 cascades for efficient exocytosis. β-arrestins act as opponents of G proteins and lead to signal termination with or without subsequent internalization. We recently demonstrated that ligand-induced internalization of MRGPRX2 requires the action of β-arrestin-1, but not of β-arrestin-2. Here, by using RNA interference, we find that both isoforms counter skin MC degranulation elicited by three MRGPRX2 agonists but not by FcεRI-aggregation. Analyzing whether this occurs through MRGPRX2 stabilization under β-arrestin attenuation, we find that reduction of β-arrestin-1 indeed leads to increased MRGPRX2 abundance, while this is not observed for β-arrestin-2. This led us speculate that β-arrestin-2 is involved in signal termination without cellular uptake of MRGPRX2. This was indeed found to be the case, whereby interference with β-arrestin-2 has an even stronger positive effect on ERK1/2 phosphorylation compared to β-arrestin-1 perturbation. Neither β-arrestin-1 nor β-arrestin-2 had an impact on AKT phosphorylation nor affected signaling via the canonical FcεRI-dependent route. We conclude that in skin MCs, β-arrestin-2 is chiefly involved in signal termination, whereas β-arrestin-1 exerts its effects by controlling MRGPRX2 abundance.

## Introduction

Mast cell (MC) activation is classically considered to be immunoglobulin E-mediated (IgE) involving the IgE receptor FcεRI and allergen (1–5). However, some basic secretagogues, including neuropeptides and drugs, are able to degranulate allergy effector cells independently of IgE, in a “pseudo allergic” or “allergoid” manner (6–15).

Mas-related G protein coupled receptor X2 (MRGPRX2) was proved to be the target of many of the agonists associated with this “second route.” Therefore, research in this field is currently very active; it will explain the pathological mechanisms underlying conditions like drug-induced anaphylaxis, atopic dermatitis, neurogenic inflammation, postoperative pain, itch, chronic spontaneous urticaria, and prurigo to enhance future diagnosis and treatment (14, 16–25).

Signaling via MRGPRX2 is still rudimentarily understood, but involves G_α*i*_ and/or G_α*q*_ to initiate cascades that culminate in degranulation (26, 27). Recent structure-function studies suggested a unique ligand-transduction mechanism in MRGPRX family receptors, as they lack many of the canonical motifs associated with signaling and ligand recognition in family A G protein-coupled receptors (GPCRs) (28, 29). Interestingly, allergic FcεRI- and pseudo-allergic pathways share the same signaling components further downstream, including extracellular signal-regulated kinase1/2 (ERK1/2), P38 and AKT, though kinetics differ between routes (26, 30–32). Both pathways also activate the guanosine triphosphatase (GTPase) Cdc42 upstream of actin cytoskeleton rearrangements, processes that involve the unconventional type I myosin 1f (MYO1F) (33).

Arrestins were first identified as “arresting” (desensitization and/or internalization) GPCR-mediated G protein signaling, which is promoted by the phosphorylation of the activated GPCR by a G protein coupled receptor kinase (GRK) (34, 35). Moreover, they can mediate their own downstream signals, including mitogen-activated protein kinases (MAPK) (36).

Four arrestin isoforms are expressed in vertebrates: while arrestin 1 and 4 is restricted to the visual system, arrestin 2 and 3 are expressed throughout the body. Arrestin 2 and 3 are also called β-arrestin-1 and−2, respectively, since they were first found to regulate β-adrenergic receptor function (37, 38). Both β-arrestin-1 and-2 are expressed by MCs (39–41). β-arrestin-1 organizes agonist-dependent MRGPRX2 internalization, and thereby desensitizes MRGPRX2 in human MCs (42). Conversely, in the mouse, it is β-arrestin-2 that regulates MC degranulation via the modification of signaling pathways (43). It remains unknown whether β-arrestin-2 influences the functional output of human MRGPRX2.

With the few exceptions, the roles of β-arrestin-1 and−2 are ill-defined in the MRGPRX2 signaling network, and barely any information is available in human skin MCs, perhaps because of the difficulty in obtaining samples for analysis. However, it is skin MCs that express MRGPRX2 at high level (40, 41, 44, 45) and are of pathophysiological significance in skin diseases. This prompted us to investigate the involvement of the β-arrestin isoforms in MRGPRX2 function in MCs of skin origin. We report that while only β-arrestin-1 negatively regulates MRGPRX2 expression, both β-arrestin-1 and β-arrestin-2 interfere with MRGPRX2 signaling and granule secretion.

## Materials and Methods

### Skin MC Purification and Culture

Foreskins from circumcisions were obtained with written informed consent of the patients or their legal guardians (26, 42, 46). The study was approved by the Ethics Committee of the Charité Universitätsmedizin Berlin (protocol code EA1/204/10, 9 March 2018) and experiments were conducted according to the Declaration of Helsinki Principles. MCs were isolated by a routine method as described (47, 48). Each mast cell preparation/culture originated from 2 to 15 donors. Briefly, skin samples were cut into strips and treated with 3.5 U/ml dispase (BD Biosciences, Heildelberg, Germany) at 4°C overnight. The epidermis was removed and the dermis was chopped and digested with an enzyme cocktail containing 1.5 mg/ml collagenase type 1 (Worthington, Lakewood, NJ, USA), 0.75 mg/ml hyaluronidase type 1-S (Sigma, Steinheim, Germany), and DNase I at 10 μg/ml (Roche, Basel, Switzerland) at 37°C in a shaking water bath for 75 min. Cells were filtrated from the remaining tissue and MCs were labeled with anti-human c-Kit magnetic microbeads for selection by an Auto-MACS separation device (both from Miltenyi Biotec, Bergisch Gladbach, Germany). MC viability was assessed by toluidine-blue staining (>99%) and purity by trypan blue purity (>98%) as well as FACS staining (double staining of KIT/FcεRI) (49, 50). Purified skin MCs from individual preparations were cultured in Basal Iscove's medium with 10% FCS (Biochrom, Berlin, Germany) the presence of SCF (100 ng/ml) (Peprotech, Rocky Hill, NJ, USA) and IL-4 (20 ng/ml) (Peprotech) freshly provided twice weekly when cultures were re-adjusted to 5 × 10^5^/ml. Cells from individual isolations (referred to as one culture) were cultured for 2–4 weeks and 3–4 d after the last addition of cytokines, cells were used for experiments. For each experiment, a different culture was used indicated by “n” provided in the accompanying figure legends.

### β-hexosaminidase Release Assay

Detection of MC degranulation by β-hexosaminidase quantification was performed as described (48, 51). Briefly, cells were treated with vehicle (spontaneous release), or challenged with codeine-phosphate at 100 μg/ml (solution prepared by the Charité pharmacy at 0.9% in water), compound 48/80 (c48/80) at 10 μg/ml (Sigma, Steinheim, Germany), substance P (SP) at 30 μM (Bachem, Budendorf, Switzerland) or anti-FcεRIα-Ab (clone AER-37, eBioscience, San Diego, CA, USA) at 0.1 μg/ml for 60 min in PAG-CM buffer (Piperazine-N,N-bis[2-ethanesulfonic acid]-Albumin-Glucose buffer containing 3 mM CaCl_2_ and 1.5 mM MgCl_2_, pH 7.4). Supernatants (SNs) were collected and the pelleted MCs were rapidly frozen with 100 μl H_2_O at −80°C. After thawing, 50 μl of SNs and lysates were incubated with 4-methyl umbelliferyl-N-acetyl-beta-D-glucosaminide (Sigma-Aldrich, Munich, Germany) solution at 5 μM in citrate buffer (pH 4.5) of the same volume and incubated for 60 min at 37°C. 100 mM sodium carbonate buffer (pH 10.7) was added to stop the reaction. Fluorescence intensity was determined at excitation at 355 nm and emission wavelength of 460 nm. Percent β-hexosaminidase release = [fluorescence intensity SN / (fluorescence intensity SN + fluorescence intensity lysate)] × 100. Net release was calculated by subtracting spontaneous release.

### RNA Interference

RNA interference in MCs was performed according to an established protocol in our laboratory employing the Accell® siRNA technology (42, 48, 52, 53) (Dharmacon, Lafayette, CO, USA). MCs were washed with Accell siRNA medium (supplemented with Non-Essential Amino Acids and L-Glutamine), plated at 1 × 10^6^/mL, and treated with 1 μM ARRB1 and ARRB2-targeting siRNA or non-targeting siRNA (each as “smart pool of 4,” E-011971-00-0050 and E-007292-00-0050 and D-001910-10-50) for 48 h, and employed for downstream analyses.

### Flow Cytometry

MCs were blocked with human AB-serum (Biotest, Dreieich, Germany) for 15 min at 4 °C and incubated with 0.15 μg/ml anti-human MRGPRX2 (clone K125H4, Biolegend San Diego, CA, USA) or PE-labeled mouse IgG2b-PE isotype control (clone eBMG2b, eBioscience, San Diego, CA, USA) for 30 min as described (51). MRGPRX2 surface expression was measured on the Facscalibur (BD Biosciences, San Jose, CA, USA) or MACSQuant (Miltenyi-Biotec, Bergisch Gladbach, Germany). Data were analyzed with the FlowJo analysis software (FlowJo LLC, Ashland, OR, USA).

### RT-qPCR

ARRB1 and ARRB2 knockdown efficiency was assessed exactly as described (51). Briefly, total RNA was isolated with Nucleo spin RNA Kit (Macherey-Nagel, Düren, Germany), and digested with RNase-free DNase (Qiagen, Hilden, Germany). Total RNA was reverse-transcribed with a first-strand synthesis kit (Invitrogen, Darmstadt, Germany) as detailed by the manufacturer, and PCR carried out with the LC Fast Start DNA Master SYBR Green kit (Roche Applied Science). Primers were 5′-CAAAGGGACCCAGTGTTCA and 5′- TTGGCCACAAACAGGTCCTT for ARRB1, 5′-CCAGGTCTTCACGGCCATAG and 5′-AGTCGAGCCCTAACTGCAAG for ARRB2. The values were normalized to the housekeeping genes β-actin, cyclophilin B, and GAPDH. The primers were CTGGAACGGTGAAGGTGACA and AAGGGACTTCCTGTAACAATGCA for β-actin, AAGATGTCCCTGTGCCCTAC and ATGGCAAGCATGTGGTGTTT for Cyclophilin B, ATCTCGCTCCTGGAAGATGG and AGGTCGGAGTCAACGGATT for GAPDH. Each ratio was contrasted against control conditions and the mean of the three determinations was used for the analysis.

### Immunoblotting

Detection of ERK1/2 and AKT phosphorylation was as described (26, 30, 48). In brief, MCs were stimulated at 5 × 10^5^/ml with c48/80 (10 μg/ml), SP (30 μM), or codeine (100 μg/ml) for 1 min, or FcεRI aggregation by AER-37 (0.1 μg/ml) for 30 min; cells kept without stimulus served as control. Samples containing the same number of cells were centrifuged and boiled in SDS-PAGE sample buffer for 10 min. Cell lysates were separated through 4–12% Bis-Tris gels (Thermo Fisher Scientific, <city>Berlin</city>, Germany), transferred to a blotting membrane and incubated with the antibodies specified. Visualization was done using a chemiluminescence assay (Weststar Ultra 2.0, Cyanagen, Bologna, Italy) and bands recorded on a chemiluminescence imager (Fusion FX7 Spectra, Vilber Lourmat, Eberhardzell, Germany).

The following primary antibodies were employed, all purchased from Cell Signaling Technology (Frankfurt am Main, Germany): anti-pAKT (1:500 dilution, #9271), anti-AKT (1:1,000 dilution, #9272), anti-pERK1/2 (1:1,000 dilution, T202/Y204, #9101), anti-ERK1/2 (1:1,000 dilution, #9102), anti-Cyclophilin B (1:10,000 dilution, #43603) and anti-β-actin (1:5,000 dilution, #4967). Goat anti-rabbit IgG peroxidase-conjugated antibody was administered as the secondary detection antibody (1:10,000 dilution, Merck, #AP132P). Quantification of the recorded signals was performed using the ImageJ software (Rasband, W.S., ImageJ, U. S. National Institutes of Health, Bethesda, Maryland, USA, https://imagej.nih.gov/ij/, 1997-2018). Individual intensity values for the detected proteins were normalized to the intensity of the respective total kinase, to cyclophilin B or to β-actin as loading controls of the same membrane.

### Statistical Analysis

Statistical analyses were performed using PRISM 9 (GraphPad Software, La Jolla, CA, USA). RM one-way ANOVA with Holm-Šídák's multiple comparisons test was performed when data were normally distributed. Friedman test with Dunn's multiple comparisons test was applied when data were not normally distributed. When the ARRB1- or ARRB2-siRNA treated groups were normalized against the non-target control group (set as 1), differences were compared with one sample *t*-test (normally distributed) or Wilcoxon signed-rank test (not normally distributed), *p* < 0.05 was considered statistically significant.

## Results

### Relative Expression of ARRB1 and ARRB2 in Skin MCs

Both β-arrestins, encoded by the ARRB1 and ARRB2 genes, were robustly expressed in skin MCs in the Fantom5 expression atlas with a mean of 105 transcripts per million (tpm) for ARRB1 and 167 tpm for ARRB2. This was above the mean of all Fantom5 samples (Supplementary Figure 1A). Expression of β-arrestins was readily detectable at protein level in our mass spectrometry-based global proteome on skin MCs (manuscript in preparation) (Supplementary Figure 1B), whereby both isoforms were also above an average MC protein by IBAQ score. Our data are in accordance with those published by Plum et al., who likewise employed a mass-spectrometry method to globally interrogate the proteome of skin MCs (54) (Supplementary Figure 1C). At the protein level, expression of β-arrestin-1 was slightly higher than that of β-arrestin-2 (Supplementary Figures 1B,C), but the differences were modest and inverted at the mRNA level (Supplementary Figure 1A). Both arrestins were also detectable by RT-qPCR (Supplementary Figure 2).

### β-arrestin-1 and β-arrestin-2 Both Interfere With MRGPRX2-Driven Degranulation

**Figure 1:**
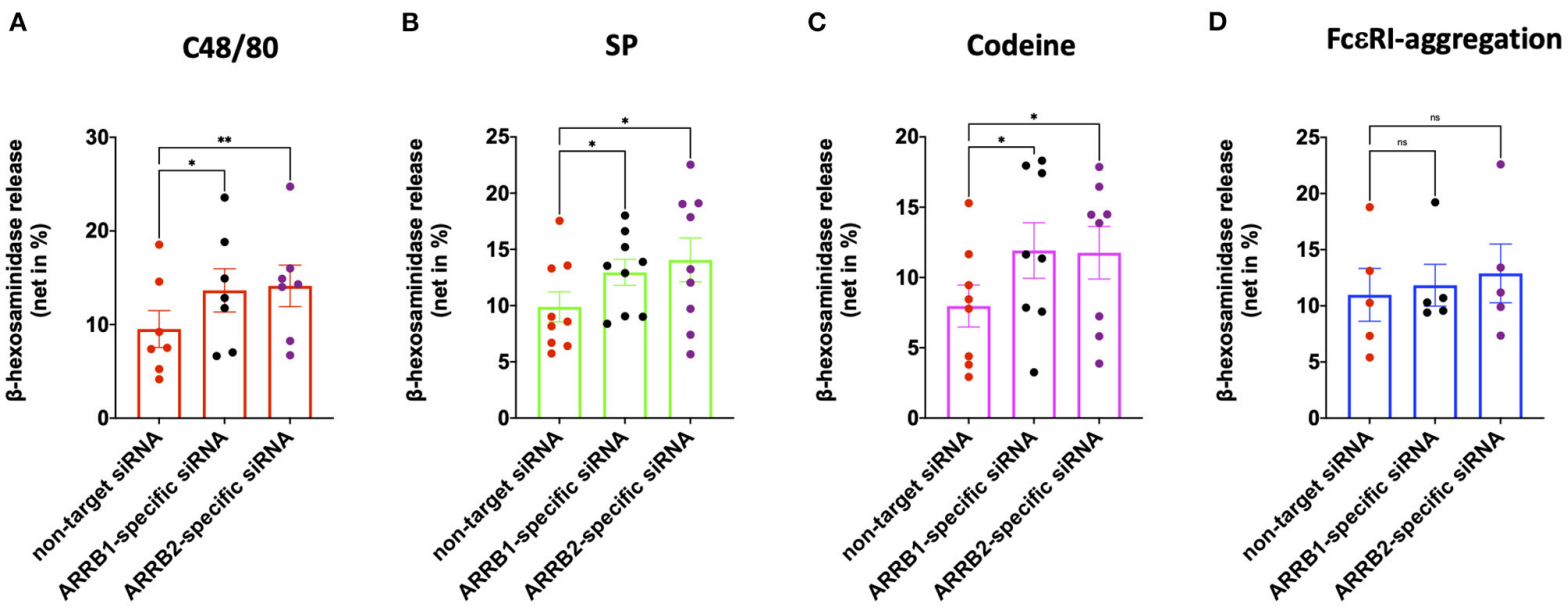
MRGPRX2 but not FcεRI-driven degranulation is regulated by β-arrestin-1 and β-arrestin-2. Human skin derived MCs were treated with ARRB1-selective, ARRB2-selective or non-target siRNA for 48 h. The cells were then stimulated with **(A)** c48/80 (10 μg/ml), **(B)** SP (30 μM), **(C)** codeine (100 μg/ml), or **(D)** FcεRI aggregation (AER-37, 0.1 μg/ml). Degranulation was determined by β-hexosaminidase release. Each dot represents an independent culture, the data shown are mean ± SEM of n=5-9. *p < 0.05, **p < 0.01, and ns, not significant.

We recently reported that β-arrestin-1 but not β-arrestin-2 is involved in MRGPRX2 internalization elicited by its ligand codeine in skin MCs (42). Here, we asked whether β-arrestin-1 or β-arrestin-2 have an impact on MRGPRX2-triggered degranulation. Both ARRB1- and ARRB2-selective siRNAs resulted in knockdown of their respective targets (Supplementary Figures 2A,B), confirming our previous report (42). Interference with β-arrestin-1 elevated secretion by all agonists employed, namely c48/80, SP and codeine (Figures 1A–C). Surprisingly, at least the same increase in exocytosis was detected upon perturbation of β-arrestin-2. In contrast, RNAi of either ARRB1 or ARRB2 had no impact on the degranulation elicited via the canonical allergic route (Figure 1D). Moreover, neither ARRB1 nor ARRB2 had an effect on spontaneous release of β-hexosaminidase (Supplementary Figure 3). We conclude that both β-arrestins act as negative regulators of MRGPRX2-initiated granule discharge, whereby both entities contribute comparably.

### β-arrestin-1, but Not β-arrestin-2, Negatively Regulates MRGPRX2 Expression

**Figure 2:**
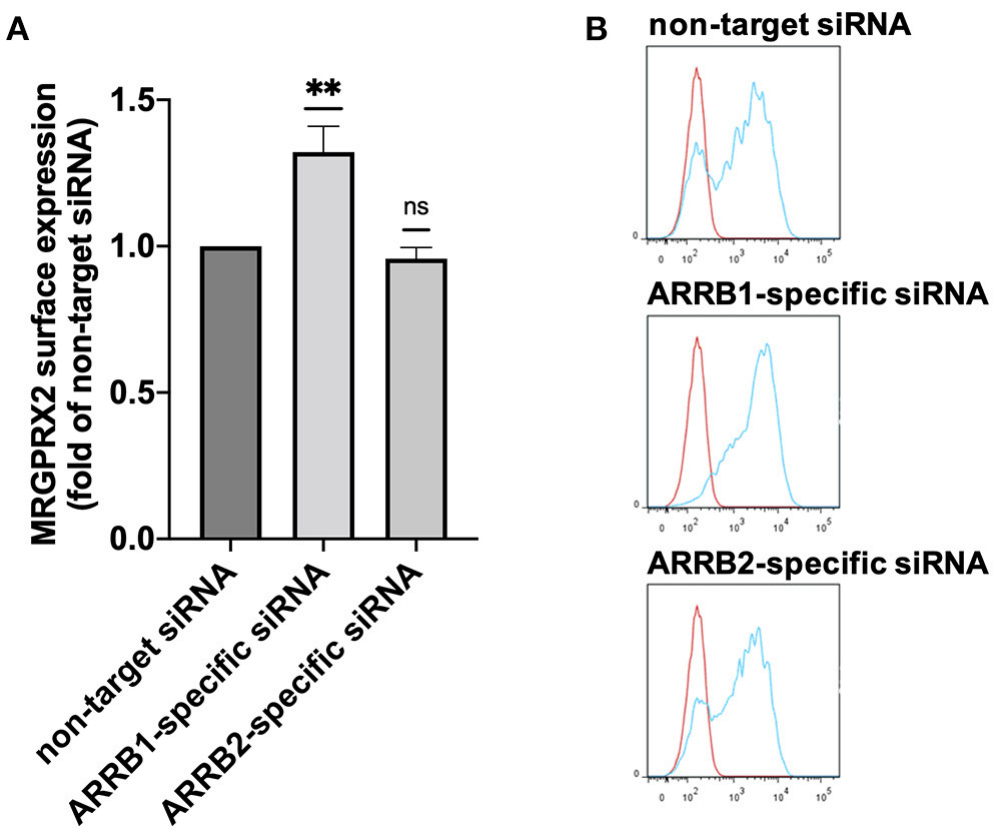
Knockdown of β-arrestin-1 promotes MRGPRX2 expression. Skin MCs were incubated with non-target siRNA, ARRB1-selective or ARRB2-selective siRNA for 48 h, MRGPRX2 cell surface expression was quantified by flow cytometry. **(A)** Net median fluorescence intensity (MFI) = (MFI MRGPRX2–MFI isotype) upon knockdown normalized to the respective non-target control (set as 1). The data are the mean ± SEM of *n* = 10, **p < 0.01, and ns, not significant. **(B)** Representative histograms of A. blue: MRGPRX2, red: isotype control.

Having found that both β-arrestins interfere with MRGPRX2-triggered degranulation of skin MCs, we asked whether β-arrestins alter MRGPRX2 cell surface expression through altered receptor dynamics. In fact, MRGPRX2 cell surface expression was enhanced by ≈40% on interference with β-arrestin-1. However, this effect was not reproduced by β-arrestin-2 perturbation under which MRGPRX2 cell surface expression remained unaltered (Figures 2A,B). Increased MRGPRX2 surface expression after ARRB1-RNAi did not result from changes in MRGPRX2 mRNA levels, which were comparable following ARRB1 perturbation (Supplementary Figure 4). In contrast, ARRB2-siRNA was associated with modest reduction of MRGPRX2-specific transcript, which did not translate to the protein at the cell surface, however (Figure 2 vs. Supplementary Figure 4). It is therefore unlikely that the increase in MRGPRX2 surface expression after ARRB1-siRNA stems from transcriptional changes, but rather from stabilization resulting from β-arrestin-1 reduction. We conclude that β-arrestin-1 affects MRGPRX2 expression by a post-transcriptional mechanism, while β-arrestin-2 does not control receptor abundance at the cell surface.

### Both β-arrestin-1, and More Strongly β-arrestin-2, Counter MRGPRX2-Mediated Phosphorylation of ERK1/2

Figure 1 showed that β-arrestin-1 and β-arrestin-2 restrict MRGPRX2's secretory competence to a similar degree, whereas Figure 2 demonstrated that only β-arrestin-1 reduction supports MRGPRX2 *expression*, calling for an alternative modus operandi in the case of β-arrestin-2. In skin MCs, both ERK1/2 and AKT are crucial to granule discharge activated via MRGPRX2 or FcεRI, as demonstrated recently by us (26). We therefore postulated that β-arrestin-2 deficiency may promote phosphorylation events downstream of MRGPRX2.

**Figure 3:**
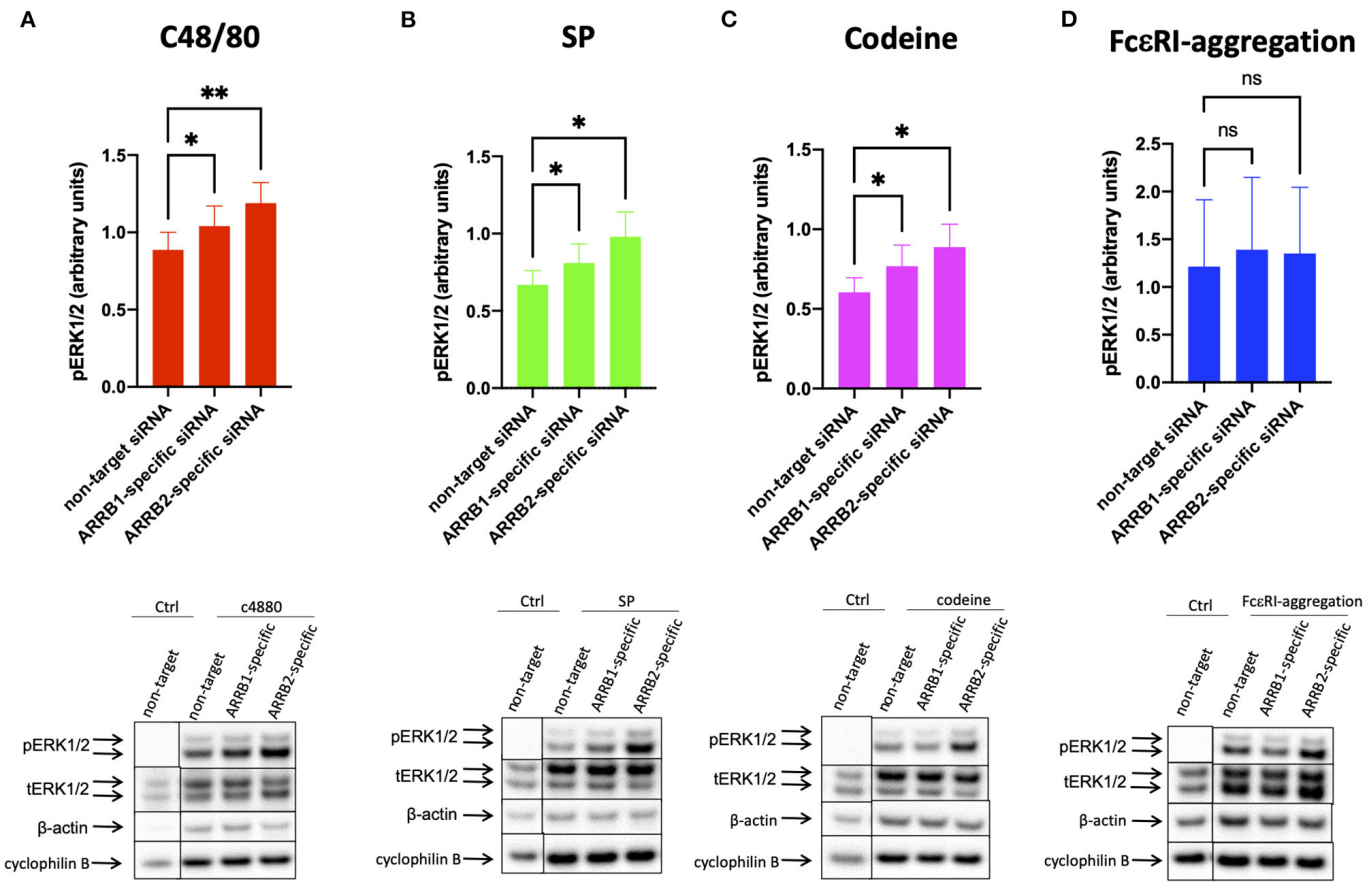
β-arrestin-1 and β-arrestin-2 interfere with MRGPRX2-mediated ERK1/2 phosphorylation. MCs were treated with ARRB1-selective, ARRB2-selective, or non-target siRNA for 48 h. The cells were then activated by **(A)** c48/80 (10 μg/ml), **(B)** SP (30 μM), **(C)** codeine (100 μg/ml) for 1 min, or **(D)** FcεRI aggregation (AER-37, 0.1 μg/ml) for 30 min, respectively. Cells with no stimulus are given for comparison. Phosphorylated (p) and total (t) ERK as well as β actin and cyclophilin B were detected and quantified consecutively on the same membranes. **(A–D)** upper panel: relative pERK normalized against tERK expression; lower panel: representative blots showing pERK and all loading controls. The data are shown as mean ± SEM of 4–12 independent experiments (individual cultures). *p < 0.05, **p < 0.01, and ns, not significant.

**Figure 4:**
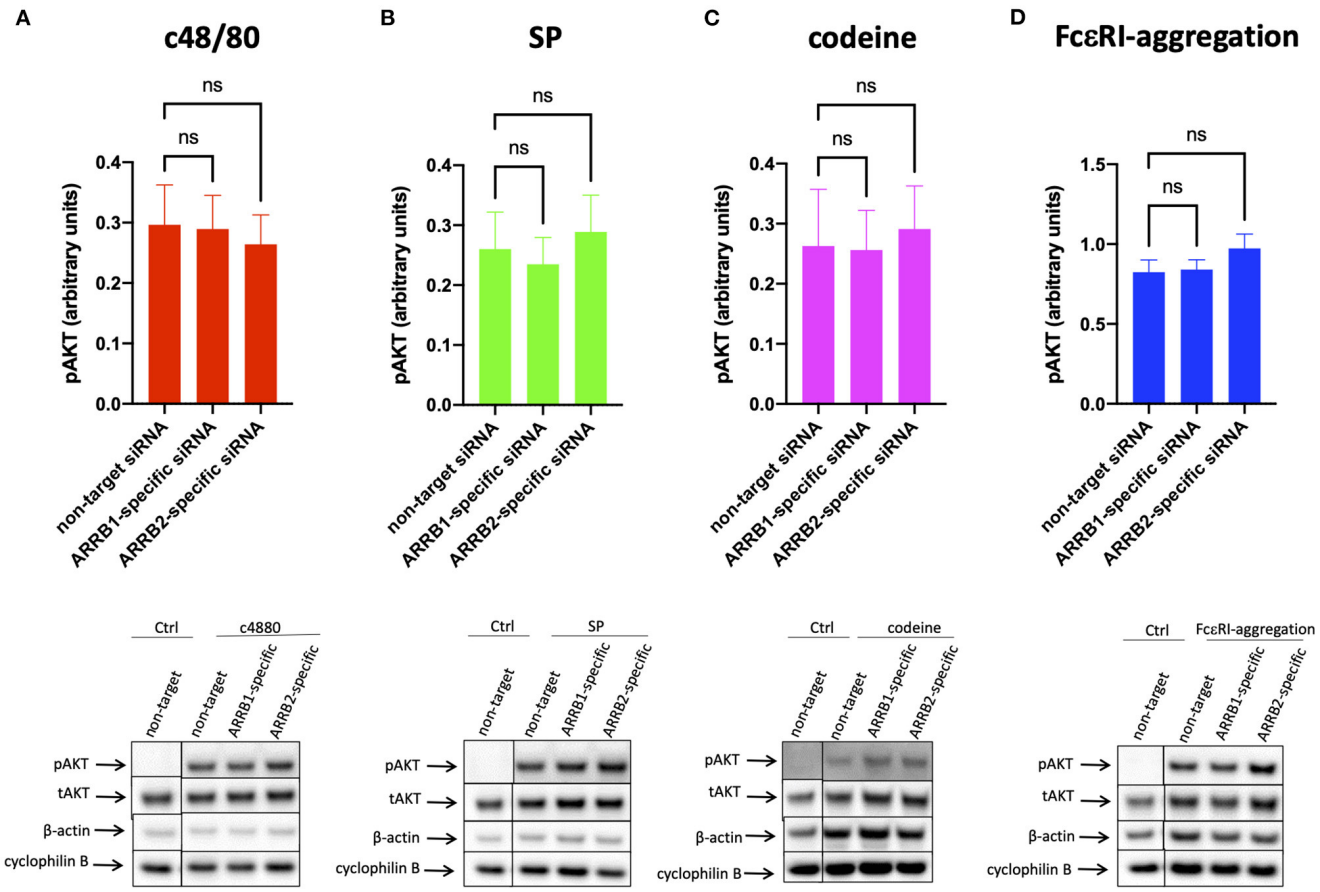
AKT phosphorylation is unaffected by β-arrestin-1 or β-arrestin-2 knockdown. MCs were treated with ARRB1-selective, ARRB2-selective, or non-target siRNA for 48 h and stimulated with **(A)** c48/80, **(B)** SP, **(C)** codeine for 1 min, or **(D)** FcεRI aggregation for 30 min exactly as indicated in Figure 3. Phosphorylated (p) and total (t) AKT and loading controls (β-actin and cyclophilin B) detected serially on the same membranes. **(A–D)** upper panel: relative pAKT normalized against tAKT expression; lower panel: representative blots showing pAKT and all loading controls. Mean ± SEM, *n* = 4–13 independent experiments, ns, not significant.

The agonists c48/80, and SP elicited ERK1/2 and AKT phosphorylation (Figures 3A,B, 4A,B) in accordance with recent findings (26, 32). While codeine was reported to act as a ligand of MRGPRX2 on skin MCs (42), codeine signaling in skin MCs was studied for the first time in the current study, duplicating the results of the other agonists (Figures 3C, 4C). In all instances, interference with β-arrestins further promoted the phosphorylation of ERK1/2. Interestingly, RNAi of ARRB2 had at least the same effect as that of ARRB1. Three different normalization strategies gave comparable results (Figure 3 lower panel and Supplementary Figure 5).

In contrast, ERK phosphorylation after FcεRI-aggregation was comparable in the experimental groups, indicating that β-arrestins do not perceptibly affect the kinase when activated by the allergic route (Figure 3D and Supplementary Figures 5D,H).

Contrary to ERK1/2, no impact on AKT phosphorylation could be detected under RNAi of ARRB1 or ARRB2, and this applied to MRGPRX2 and FcεRI alike (Figure 4 and Supplementary Figure 6).

Collectively, the data imply that β-arrestin-2 is at least as potent at MRGPRX2 signal termination as β-arrestin-1 even though reduced receptor expression can have a contributory role only in the case of β-arrestin-1. Therefore, MRGPRX2 downregulation is not a prerequisite to signal termination, and β-arrestin-2 regulates MRGPRX2 function also in human MCs.

## Discussion

There are two equally potent routes regulating MC degranulation in skin MCs, the canonical one via FcεRI/IgE plus allergen and the more recently uncovered pathway relying on MRGPRX2 and one of its numerous agonists. While other GPCRs can elicit degranulation in some MC subsets, including the complement receptors C5AR, and C3AR receptors (6), they typically do so less potently, at least in skin MCs, whereas MRGPRX2 can induce up to 60% of granule discharge in cutaneous MCs in a donor-dependent manner (42, 55). The attraction of MRGPRX2 over other GPCRs to explain clinically significant phenomena also lies in the number of ligands: While other MC-expressed GPCRs are targeted by only one or few entities, MRGPRX2 responds to several hundred already uncovered molecules, and further agonists keep being reported (6, 12, 41, 56). Ligands encompass endogenous and exogenous molecules, enabling MCs to respond to danger signals from the inside or the outside. Even though MRGPRX2 has low affinity for most of them, low concentrations of different agonists act in a complementary manner, and so do MRGPRX2 and the FcεRI pathways, which likewise enhance each other; this potentially gives rise to a large number of possible combinations *in vivo* (57).

It is therefore important to understand the molecular underpinnings of MRGPRX2 function and the associated intracellular signaling components. In the current study, we explored the roles of β-arrestins as elements of the MRGPRX2 signaling network in skin MCs. β-arrestins are intracellular proteins best known for their function to desensitize GPCRs (58–60). They act by signal termination and internalization of GPCRs although they also signal on their own and influence processes by serving as docking platforms; this gives rise to complex feedback and feedforward loops, the final consequences of which are unpredictable (61).

How β-arrestins contribute to MRGPRX2 signaling is poorly understood. The first paper investigating receptor phosphorylation and desensitization concluded that MRGPRX2 is resistant to desensitization and internalization (62). The ligand employed by that study was cathelicidin (LL-37), which now seems to act as a G protein biased ligand (i.e., a ligand not recruiting β-arrestin), even though this issue is not completely clarified (63). With the use of other agonists, it became clear that MRGPRX2 is in fact desensitized and internalized just like most other GPCRs. This is found for neuropeptides like SP, drugs like opioids, and synthetic agonists like c48/80 (41, 64, 65). In fact, the so-called TANGO (transcriptional activation following arrestin translocation) assay uses the property of β-arrestin activation as readout, and is employed in MRGPRX2 studies, e.g., to identify new agonists (41) or to distinguish between balanced and biased ligands (64–66).

We recently confirmed that c48/80, SP and codeine are balanced ligands in skin MCs whose binding elicits not only degranulation but also receptor internalization (42). In the same study we found that it is only β-arrestin-1 that contributes to ligand-driven MRGPRX2 internalization in these cells. In contrast, Roy et al. reported that β-arrestin-2 was the major participant in several processes triggered via Mrgprb2 in the mouse, suggesting differences between murine Mrgprb2 and human MRGPRX2 (43). In fact, the two receptors share only 53% sequence identity and Mrgprb2 has a much lower affinity for most agonists than its human counterpart (13, 14, 67).

Since β-arrestin-1 (but not β-arrestin-2) was found to be involved in ligand-dependent MRGPRX2 internalization, we hypothesized that β-arrestin-1 is also the isoform chiefly involved in signal termination preceding internalization. In support of this hypothesis was our current finding that β-arrestin-1 interferes with MRGPRX2 cell surface expression in the absence of specific ligand (Figure 2), suggesting regulation of MRGPRX2 dynamics also in the steady state. Contrary to our expectation, we found that both β-arrestins perturb MRGPRX2 functional competence, however. In fact, cells with reduced β-arrestin-1 or β-arrestin-2 displayed similar increase in granule discharge (Figure 1). For β-arrestin-1-specific-RNAi this could be explained by the increased amount of MRGPRX2 to be engaged in signal transduction (by higher presence at the cell surface), while an alternative mechanism had to be envisaged for β-arrestin-2.

The two β-arrestins share 70–80% sequence identity and have similar three-dimensional structures (58, 60). Desensitization by these regulatory proteins is initiated by binding to GRK-phosphorylated GPCRs, mostly in the longer cytoplasmic tail. Though both exert similar functions and can bind clathrin and its adaptor protein 2 (AP2), through which coated pit formation and GPCR internalization are enabled (58, 59), studies with cultured cells and knockout mice revealed that β-arrestin-1 and−2 are not functionally redundant (68). In fact, β-arrestin-2 has higher affinity for many GPCRs, though some GPCRs preferentially recruit β-arrestin-1 (58).

We assume that the two β-arrestins fulfill distinct functions in skin MCs, as has been reported for the GPCR C3aR, whereby β-arrestin-2 was involved in desensitization and internalization, while β-arrestin-1 was, rather unexpectedly, found as a positive regulator of degranulation in the MC line LAD2 (39). This is reminiscent of what was reported in murine MCs for Mrgprb2, where β-arrestin-2 was also the entity involved in desensitization (43). In skin MCs, however, this seems different since it is β-arrestin-1 that drives internalization. This further underlines the differences between human skin MCs and MC lines as well as between human and murine MRGPRX2/Mrgprb2 (69–71).

Since β-arrestin-2 attenuated MRGPRX2 initiated degranulation as potently as β-arrestin-1, though only the latter acted via altered MRGPRX2 expression, this provided a strong argument for β-arrestin-2's role in signal termination without cellular uptake. In fact, this could eventually be proven.

Interference with β-arrestin-2 or β-arrestin-1 increased pERK1/2 upon MRGPRX2 ligation while leaving signaling by FcεRI unperturbed. Effects tended to be stronger for ARRB2- compared to ARRB1-siRNA, highlighting they were uncoupled from receptor internalization. We recently reported that in human skin MCs, both ERK1/2 and phosphatidylinositol 3 kinase (PI3K) form part of the degranulation machinery (26). PI3K is well-established as an important module regulating granule discharge elicited via FcεRI (72), and more recently confirmed for MRGPRX2 (73, 74). In the current study, the PI3K module seemed to be largely unaffected by β-arrestin abundance. We speculate that this is related to the fact that PI3K strictly depends on G_α*i*_ activation, while ERK1/2 appears to involve the upstream action of G_α*i*_ or G_α*q*_ (26). While ERK1/2 was recently uncovered as a major component of granule discharge in human MCs, it does not appear to have this role in the mouse (26, 71, 72). In the present study, ERK1/2 was the kinase affected by β-arrestin levels associating the enhanced secretory output with a precise molecular event. In contrast, β-arrestins can initiate signaling cascades on their own, and especially ERK activation in the cytoplasm is a frequent event (36). For example, it was reported that β-arrestin-2 and G protein-mediated ERK activation exhibited differential kinetics in angiotensin II type 1A receptor, whereby G Protein-mediated ERK activation occurred quickly, while β-arrestin-2-dependent activation was slow (75). This later β-arrestin-initiated phosphorylation of ERK does not seem to be a major component of MRGPRX2 activation in skin MCs, however. Rather, our time-courses revealed that ERK phosphorylation is swift, the signal reaching a peak after 1 min and declining thereafter to nearly baseline after 30 min (32). The absence of prolonged or biphasic signaling implies that in our system, β-arrestin does not discernibly activate ERK (or other kinases) on its own. In fact, in addition to ERK, we did not detect any reduction in p38 or AKT phosphorylation after either ARRB1- or−2selective KD. A possible though speculative explanation may be that the coupling to G proteins can influence the subsequent role of β-arrestin signaling. In fact, the activation of angiotensin II type 1A receptor depends more strongly on the Gq/11 (76), while Gi is the most significant G protein activated downstream of MRGPRX2 in skin MCs (26). Another possibility is that the ratios between β-arrestins and other relevant signaling components differ in MCs compared to other cells and cell lines. Together, the current interpretation is that β-arrestins primarily act as signal terminators rather than as activators of own signaling cascades in skin MCs, at least for the kinases investigated in the present study. It should be interrogated in the future whether alternative cascades are induced by β-arrestins in skin MCs following activation of MRGPRX2 by different ligands and other GPCRs.

One limitation of our study is the incomplete nature of the RNAi technique. Indeed, reduction by about 50% may have different consequences for one isoform vs. the other, where anything between nullification of function (in the case of function-restricting expression at baseline) to virtually no effect (if the protein is expressed at overabundant or saturating levels) is imaginable. Even though both isoforms seem to be similarly expressed in MCs (Supplementary Figure 1), one isoform may still be functionally more active and/or knockdown efficiency at the protein level may differ between subsets. Ideally, knockdown studies should be accompanied by selective inhibitors targeting one or the other arrestin at the functional level. The future identification of such substances will hopefully allow this type of studies and help unraval their distinct roles in various processes of different cells, including MCs. Inhibitor development is currently underway (77), but with IC50 values of 19.1 and 15.6 μM for β-arrestin-1 and β-arrestin-2, the substance currently available (Barbadin) does not differentiate between the isoforms.

Collectively, with the above caveats in mind, the functions of β-arrestin-1 and β-arrestin-2 seem to differ in skin MCs. While β-arrestin-1 is the entity regulating MRGPRX2 cell surface expression, β-arrestin-2 has no effect on receptor abundance. Despite this difference, β-arrestin-1 and β-arrestin-2 have similar roles in skin MC degranulation and signaling. In fact, interference with either will support degranulation and ERK1/2 activation, as consistently revealed for three agonists. Therefore, even though engaging distinct mechanisms, β-arrestin-1 and β-arrestin-2 similarly interfere with MRGPRX2-triggered exocytosis in cutaneous MCs.

## Data Availability Statement

The original contributions presented in the study are included in the article/Supplementary Material, further inquiries can be directed to the corresponding author.

## Ethics Statement

The studies involving human participants were reviewed and approved by the Ethics Committee of the Charité Universitätsmedizin Berlin. Written informed consent to participate in this study was provided by the participants' legal guardian/next of kin.

## Author Contributions

MB: conceptualization and project administration. ZW, ZL, GB, and KF: investigation. MB, ZW, KF, GB, and ZL: data curation. MB, ZW, and ZL: writing—original draft preparation and visualization. MB, ZW, GB, KF, and TZ: writing—review and editing. MB and TZ: supervision and funding acquisition. All authors have read and agreed to the published version of the manuscript.

## Funding

This work was supported by the Deutsche Forschungsgemeinschaft (BA-3769/4-1) to MB. ZW was funded by scholarships from CSC and Charité. ZL was funded by a scholarship from CSC. The study also received funding from ECARF (European Center for Allergy Research Foundation) to TZ.

## Conflict of Interest

The authors declare that the research was conducted in the absence of any commercial or financial relationships that could be construed as a potential conflict of interest.

## Publisher's Note

All claims expressed in this article are solely those of the authors and do not necessarily represent those of their affiliated organizations, or those of the publisher, the editors and the reviewers. Any product that may be evaluated in this article, or claim that may be made by its manufacturer, is not guaranteed or endorsed by the publisher.
