# Supplementary material for: β-arrestin-1 and β-arrestin-2 Restrain MRGPRX2-Triggered Degranulation and ERK1/2 Activation in Human Skin Mast Cells
Source: Front Allergy. 2022 Jul 15;3:930233. doi: 10.3389/falgy.2022.930233 (PMC9337275; doi:10.3389/falgy.2022.930233)
Supplement: Supplementary file 1 [file Data_Sheet_1.pdf]

Supplementary Material

Supplementary Figures

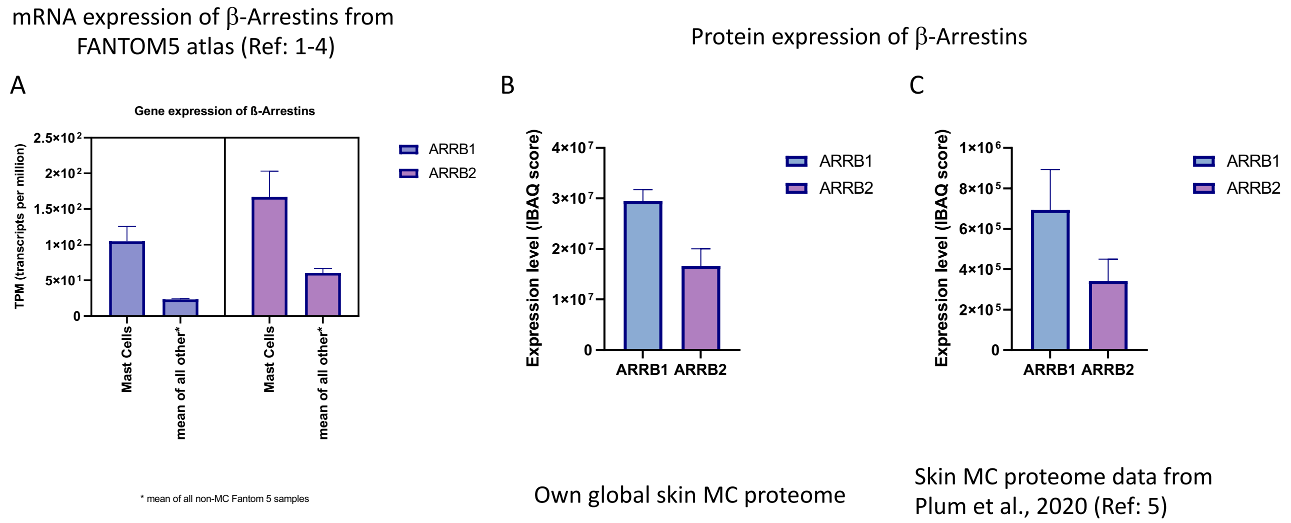

Supplementary Figure 1. Relative expression of ARRB1 and ARRB2 in skin MCs

A) Relative gene expression of β-arrestins was retrieved from the FANTOM5 atlas (1-4). Expression levels (tpm = transcripts per million) of ARRB1 and ARRB2 in the 6 non-stimulated MC samples (mean ± SEM) are put side by side to an average non-MC. B, C) Intensity-based absolute quantification (IBAQ) values represent β-arrestin-1 and -2 protein abundance in skin mast cells. B) the data are from an unpublished own mass spectrophotometric proteomics study [manuscript in preparation]. C) IBAQ Data were extracted from a published proteome study on skin MCs (5).

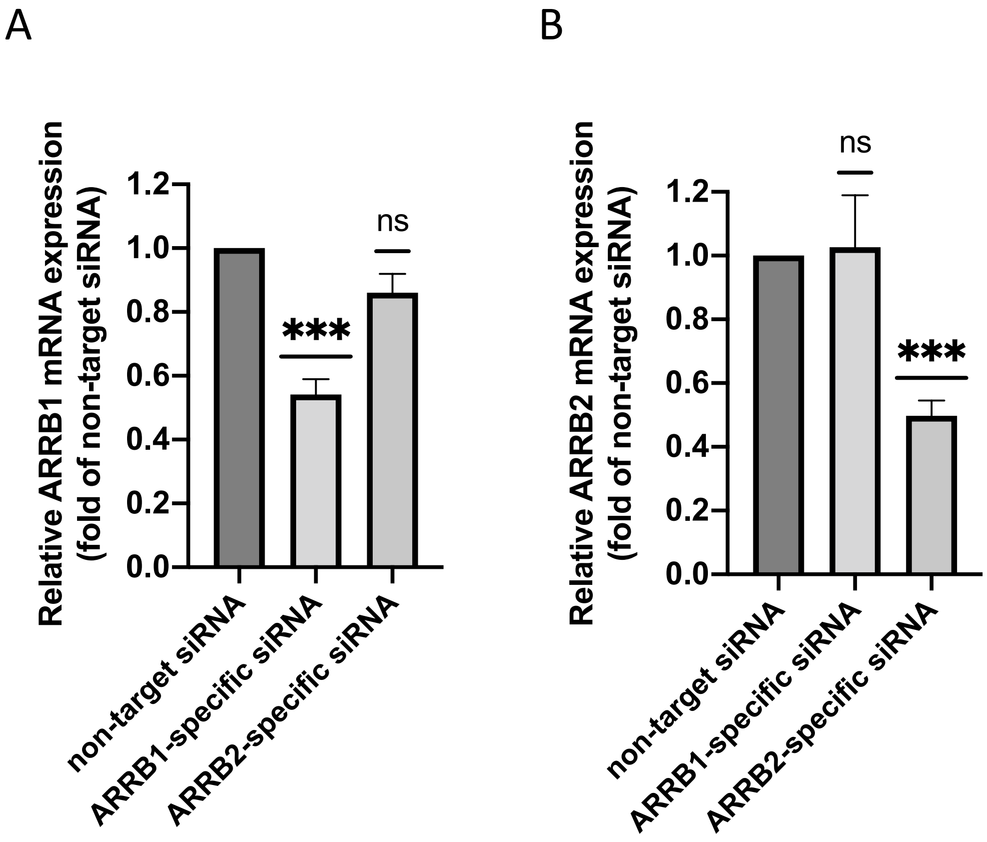

Supplementary Figure 2. ARRB1 and ARRB2 knockdown efficiency

Human skin MCs were treated with non-target siRNA or ARRB1-, ARRB2-specific siRNA for 48h. mRNA expression of ARRB1 and ARRB2 were quantified by RT-qPCR. Mean ± SEM, n=5. *** $p < 0.001$, ns: not significant.

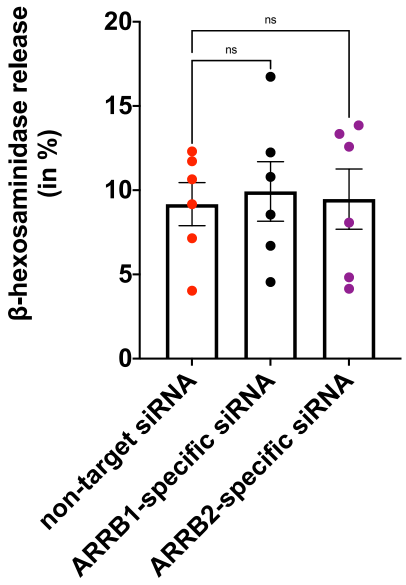

Supplementary Figure 3. Mast cell spontaneous degranulation is not altered by β-arrestin-1 and β-arrestin-2 siRNA

Human skin derived MCs were treated with ARRB1-selective, ARRB2-selective or non-target siRNA for 48h. Spontaneous degranulation (in the absence of stimuli) was determined by β-hexosaminidase release. Each dot represents an independent culture, the columns show the mean ± SEM of n=6. ns: not significant.

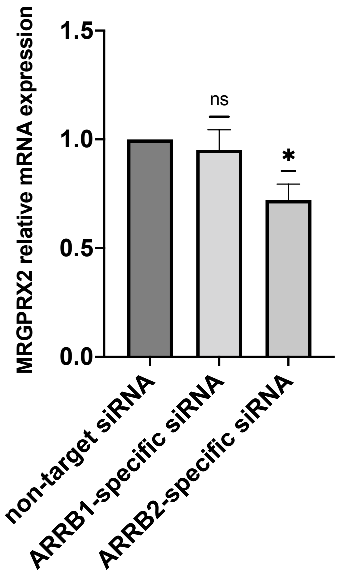

Supplementary Figure 4. MRGPRX2 mRNA expression after β-arrestin-1 and β-arrestin-2 siRNA treatment

Human skin derived MCs were treated with ARRB1-selective, ARRB2-selective or non-target siRNA for 48h. MRGPRX2 mRNA expression was determined by RT-qPCR. The data are the mean ± SEM of n=9. * $p < 0.05$, ns: not significant.

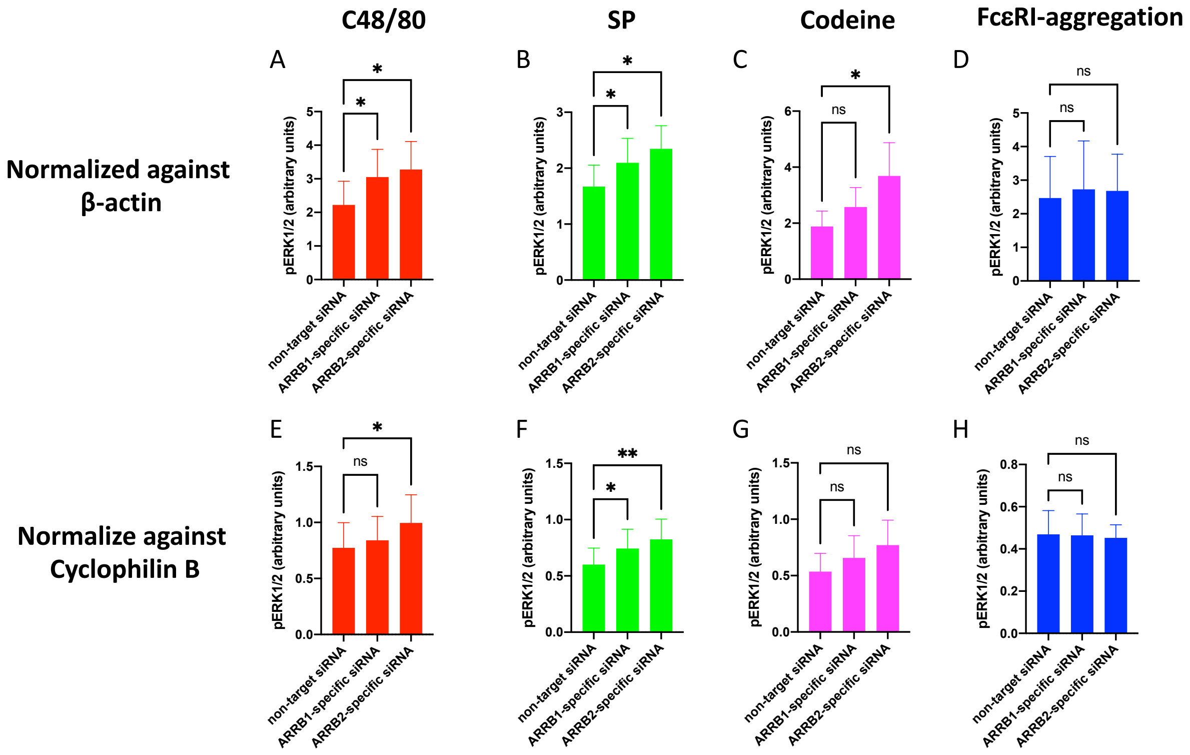

Supplementary Figure 5. ERK phosphorylation normalized against β-actin and cyclophilin B

MCs were treated as specified in Figure 3. ERK phosphorylation was quantified and normalized against the expression of A-D) β-actin and E-H) cyclophilin B of the same membrane. The data are shown as mean ± SEM of 4-13 independent experiments (individual cultures). * $p < 0.05$, ** $p < 0.01$, ns: not significant.

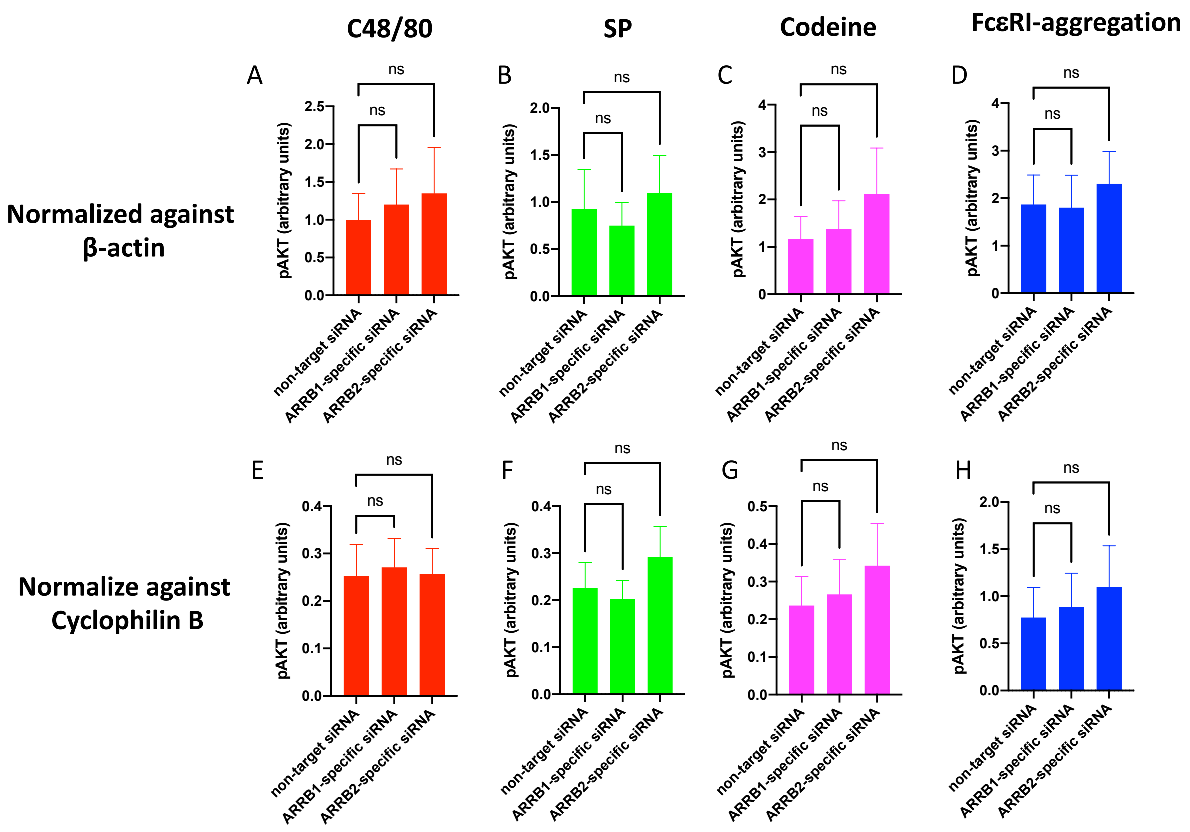

Supplementary Figure 6. AKT phosphorylation normalized against β-actin and cyclophilin B

MCs were treated as specified in Figure 4. AKT phosphorylation was quantified and normalized against the expression of A-D) β-actin and E-H) cyclophilin B of the same membrane. The data are shown as mean ± SEM of 4-12 independent experiments (individual cultures). ns: not significant.

References

1. Consortium F, the RP, Clst, Forrest AR, Kawaji H, Rehli M, et al. A promoter-level mammalian expression atlas. Nature. 2014;507(7493):462-70.

2. Arner E, Daub CO, Vitting-Seerup K, Andersson R, Lilje B, Drablos F, et al. Transcribed enhancers lead waves of coordinated transcription in transitioning mammalian cells. Science. 2015;347(6225):1010-4.

3. Noguchi S, Arakawa T, Fukuda S, Furuno M, Hasegawa A, Hori F, et al. FANTOM5 CAGE profiles of human and mouse samples. Sci Data. 2017;4:170112.

4. Severin J, Lizio M, Harshbarger J, Kawaji H, Daub CO, Hayashizaki Y, et al. Interactive visualization and analysis of large-scale sequencing datasets using ZENBU. Nat Biotechnol. 2014;32(3):217-9.

5. Plum T, Wang X, Rettel M, Krijgsveld J, Feyerabend TB, Rodewald HR. Human Mast Cell Proteome Reveals Unique Lineage, Putative Functions, and Structural Basis for Cell Ablation. Immunity. 2020;52(2):404-16 e5.
