# Supplementary figures and images for: β-arrestin-1 and β-arrestin-2 Restrain MRGPRX2-Triggered Degranulation and ERK1/2 Activation in Human Skin Mast Cells
Source: Front Allergy. 2022 Jul 15;3:930233. doi: 10.3389/falgy.2022.930233 (PMC9337275; doi:10.3389/falgy.2022.930233)

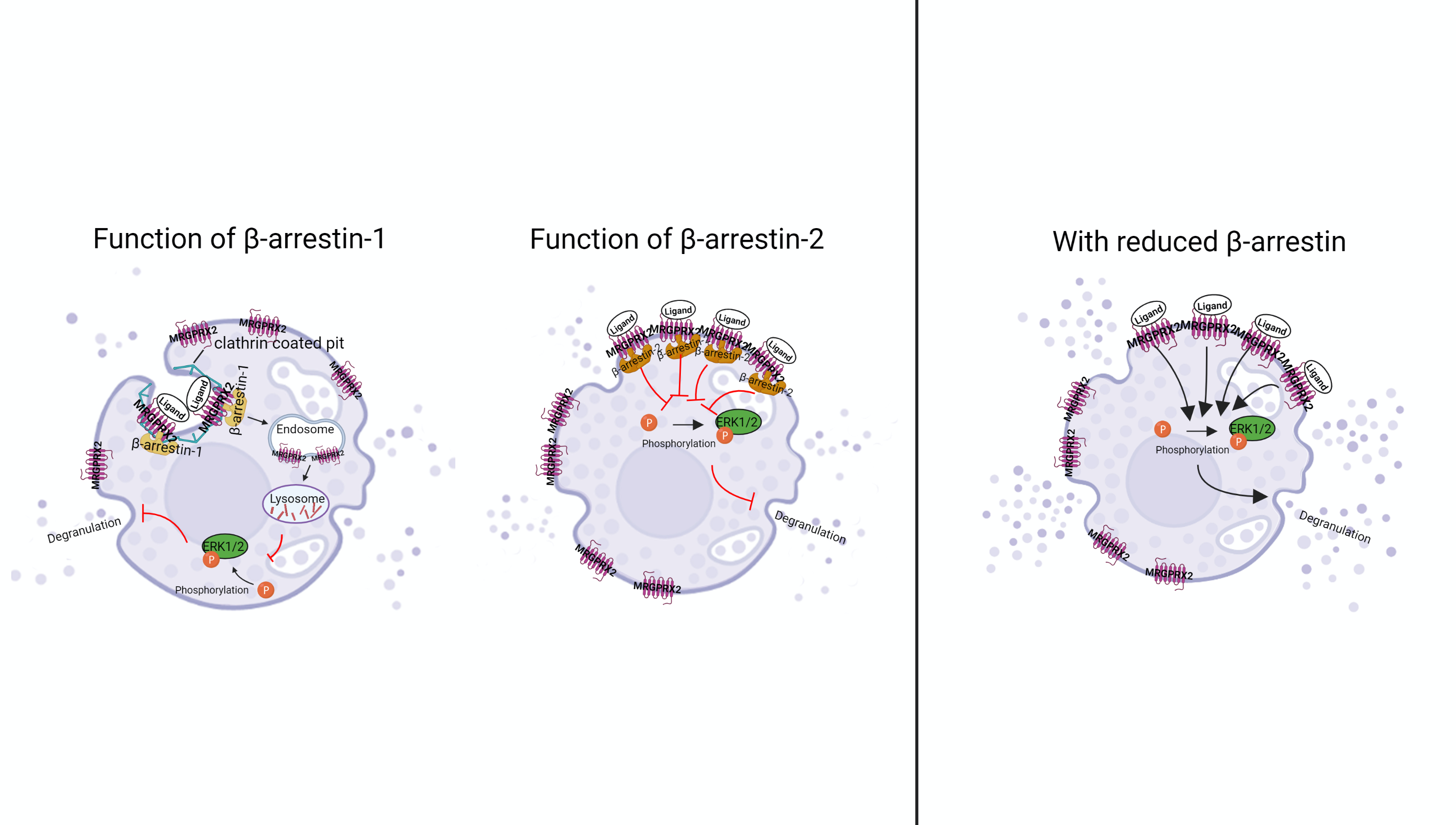

Supplement: Supplementary file 2 [file Image_1.TIFF]
